# Supplementary material for: A nanophotonic laser on a graph
Source: Nat Commun. 2019 Jan 15;10:226. doi: 10.1038/s41467-018-08132-7 (PMC6333791; doi:10.1038/s41467-018-08132-7)
Supplement: Supplementary file 1 — Supplementary Information [file 41467_2018_8132_MOESM1_ESM.pdf]

Supplementary Information: A nanophotonic laser on a graph

Michele Gaio, Dhruv Saxena, Jacopo Bertolotti, Dario Pisignano, Andrea Camposeo, and Riccardo Sapienza*

* r.sapienza@imperial.ac.uk

Supplementary Note 1: Estimation of scattering loss at a node

Finite-difference time-domain (FDTD) simulations (using Lumerical Solutions) were performed to estimate the scattering loss at nodes of the photonic network lasers. Since the most common degree (D) in the networks was 4, we modelled a node with an X-crossing (schematic shown in Supplementary Figure 1). To calculate the scattering loss, a guided mode was injected along a branch towards the node and the total power transmitted (or reflected) was measured through all branches.

Supplementary Figure 1 shows the loss in a X-branch node as a function of angle, for the two lowest order modes. In these simulations, the structure was free-standing in air, and the fibre diameter was 500 nm, index was 1.5 and the mode wavelength was 600 nm. Loss was calculated for the configuration where the two fibres were just touching at the node, and when the two fibres were merged together. As shown in Supplementary Figure 1, the average loss is below 0.15 when the fibres are merged and below 0.04 when the fibres are touching.

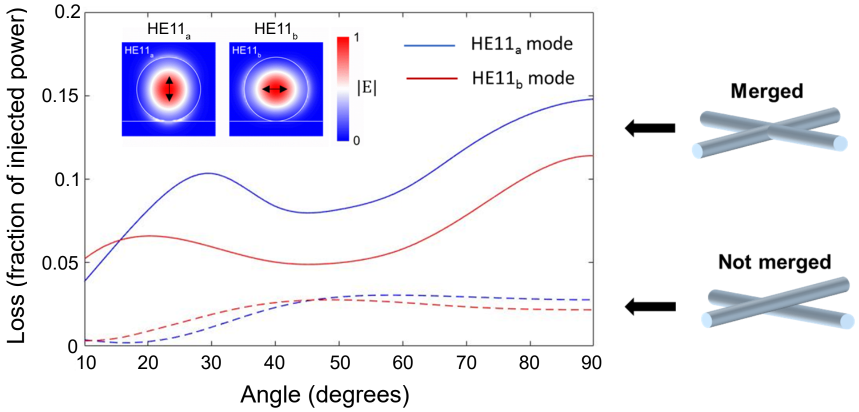

Supplementary Figure 1. **Scattering loss in a X-branch**. Loss in a X-branch is calculated for the two lowest order modes (HE11 modes of different polarizations) as a function of angle. The electric field profiles of these modes is shown in the inset. The data was calculated from 3D FDTD simulations, in which the diameter of the fibres was 500 nm, index was 1.5 and the mode wavelength was 600 nm. The fibres were either merged or not merged, as indicated.

Supplementary Note 2: Wave equation on a graph

A graph is a collection of nodes $i = 1..n$, some pairs connected by edges $r = 1..N$. We solve the 1D scalar wave equation:

$$\frac{\partial^2 E(x,t)}{\partial t^2} - v^2 \frac{\partial^2 E(x,t)}{\partial x^2} = 0 \tag{1}$$

where $v = c/n$ is the speed of light. On each edge r of the graph the solutions of the wave equation in the Helmholtz form:

$$\nabla^2 E(x) + k^2 E(x) = 0 \tag{2}$$

with $k = \omega/v$, are of the form:

$$E_r(x,k) = B_r^+ exp(\imath kx) + B_r^- exp(-\imath kx) \tag{3}$$

A graph with N edges is described by N of such equations. The $2N$ coefficients $X_{1..2N} = \{B_{1..N}^+, B_{1..N}^-\}$ are defined by the behaviour at the nodes. As boundary conditions we impose the continuity of the solution at the node position x_i:

$$E^r(x_i) = E^s(x_i) \tag{4}$$

for all edges r, s which are connected to the node i, giving overall $2N - n$ equations, and

$$\sum_r \left.\frac{dE(x)}{dx}\right|_{x=x_i} = 0 \tag{5}$$

for all connected edges r at each node (n equations), with the positive direction of x being consistently in-going or out-going for all terms. This boundary conditions are equivalent to those of the graph Laplacian operator defined as $\nabla^2 = A - D$, where A is is the adjacency matrix describing the graph and D is the connectivity degree diagonal matrix of the graph. Given a certain k, such conditions might be put in a $2N \times 2N$ sparse matrix $M(k)$, such that:

$$M(k) \cdot X = 0 \tag{6}$$

The eigenvalues of the system are given by the condition:

$$\det(M(k)) = 0 \tag{7}$$

Given the high sparsity of the matrix M, the eigenvalues can be efficiently computed even for very large number of nodes. Numerically, the condition number C of $M(k)$ is estimated (`condest` function in MATLAB) on a grid of the complex k region of interest to identify k (maxima of C) for which the matrix is singular. Subsequently, each located guess for k is optimized (simplex search) until convergence.

Supplementary Note 3: Estimate of threshold and inverse participation ratio

The inverse participation ratio (IPR) is defined as:

$$\text{IPR} = L \frac{\int |E|^4 dl}{\left(\int |E|^2 dl\right)^2}, \tag{8}$$

with L the total length of the network. From the definition of Q value:

$$Q = \frac{\text{Re}(k)}{2|\text{Im}(k)|} = \omega \cdot \frac{\text{STORED ENERGY}}{\text{LOSSES PER CICLE}} = \omega \cdot \frac{\int u(l) dl}{\sum_{\text{perimeter}} S}, \tag{9}$$

with u the energy density and S the pointing vector:

$$u = \epsilon_0 |E|^2, \qquad S = c\epsilon_0 |E|^2 \tag{10}$$

We compute these quantities assuming that a mode electric fields $E(x, y)$ is exponentially decaying radially from the centre of the network which occupy a disc or radius R:

$$|E|^2(r) \simeq e^{-r/\xi} \tag{11}$$

with ξ the localization length. This integrals are computed in the limit of the radius of the network $R \gg \xi$ (localized regime), and assuming uniform density of the networks nodes, so that the integrals over the network (dl) can be computed over the plane in which it is embedded ($dl = \alpha dx dy$), with $\alpha = \frac{L}{2\pi R^2}$, while the summation is computed defining the density of output channels over the perimeter $\beta = \frac{\#(\text{output channles})}{2\pi R}$, so that:

$$\mathrm{IPR} = \left(\frac{R}{2\xi}\right)^2 \tag{12}$$

$$Q = \frac{\mathrm{Re}(k)\xi^2\alpha}{R\beta e^{-R/\xi}} \tag{13}$$

from which:

$$-\,\mathrm{Im}(k) = \frac{R\beta e^{-R/\xi}}{2\xi^2\alpha}. \tag{14}$$

The parameter α and β are obtained directly from the network structure and depend on the density of nodes and network nodes degree ($\beta/\alpha \simeq 0.5$).

Supplementary Note 4: Spectral evolution when increasing the pump energy

Supplementary Figure 2 shows the evolution of the lasing spectrum as a function of the pump energy. Above the lasing threshold many sharp spikes develop. When the pump energy is increased even further more modes start to lase while the already present lasing peaks simply grow in intensity with minimal spectral shift. This is consistent with lasing from localized modes, which are expected to be stable both spatially and spectrally.

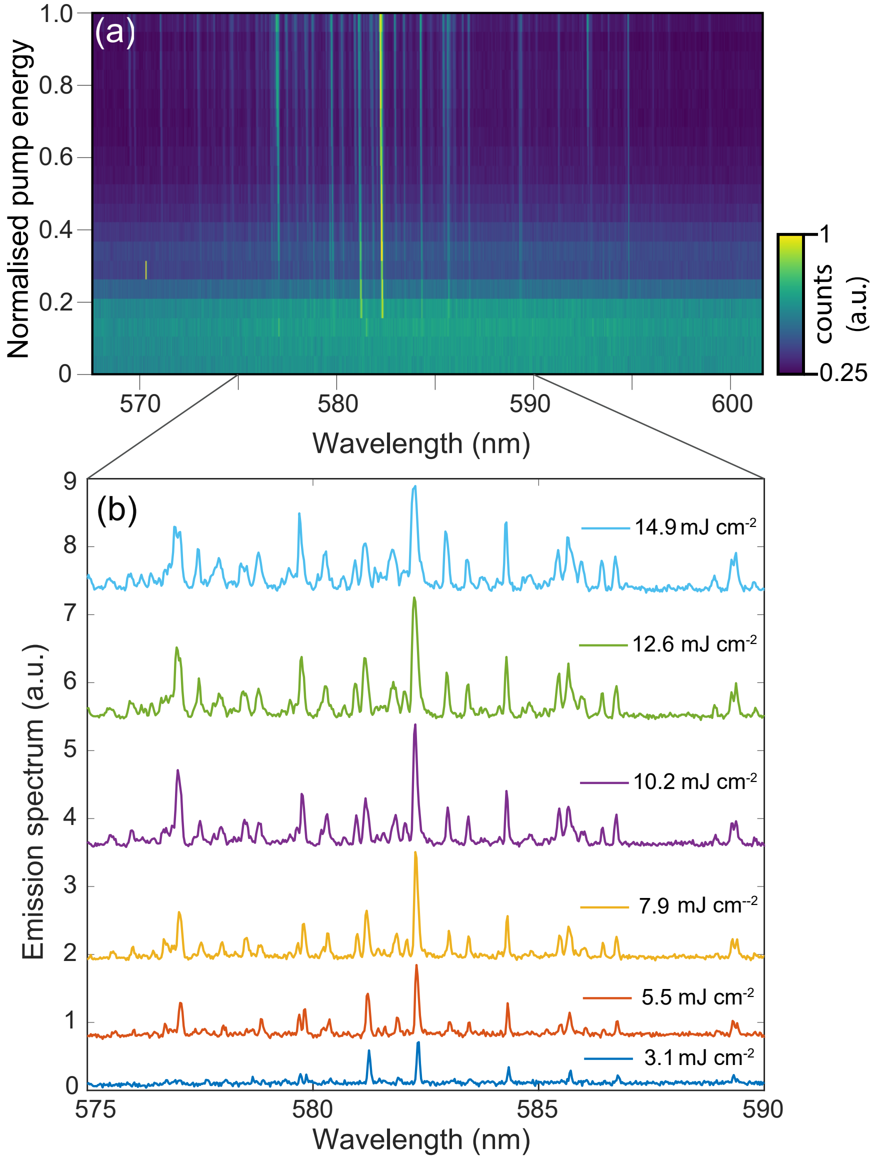

Supplementary Figure 2. **Spectral evolution**. Emission spectrum versus pump power. Upon increasing pump energy lasing peaks develop which are stable in their frequency. In panel (a) each spectrum is normalized to its maximum to allow for comparison, while panel (b) is a zoom in showing the emission spectrum at six increasing powers.

Supplementary Note 5: Spectral stability

The pulse to pulse stability is studied above threshold. Supplementary Figure 3 plots 100 spectra taken at the same nominal pump energy (P=15 mJ cm^{-2}) with single pulse excitation. The spectrum is very stable with only a minor 10-15 % fluctuation induced by the pump laser intensity fluctuations.

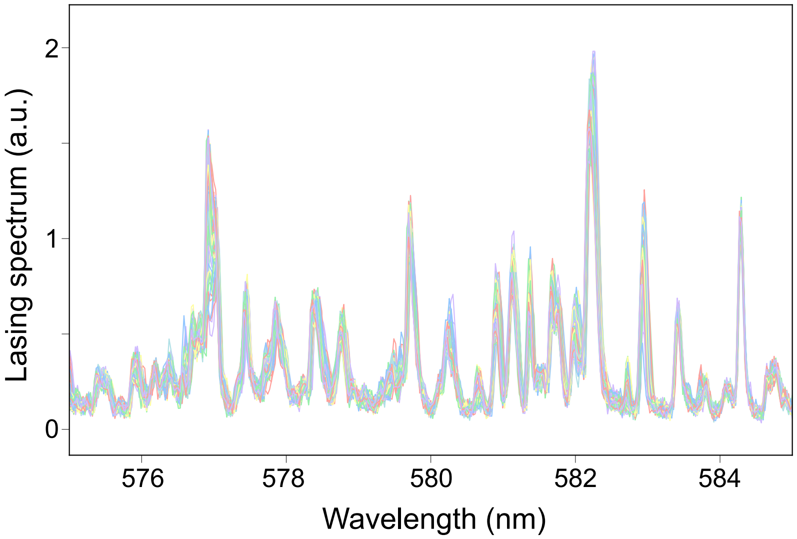

Supplementary Figure 3. **Spectral stability**. Repeated measurements of the lasing spectrum, above threshold and for the same nominal pump energy of 15 mJ cm^{-2}.

Supplementary Note 6: Mode overlap

The lasing modes in the network occupy different sets of network links. This is evident in Supplementary Figure 4a, which shows the three different modes in Figure 2a-c of the main manuscript (in red, blue and green, respectively) overlayed on top of each other. For a more detailed comparison, we compared hyperspectral images of 32 different modes by numerically computing the correlation between them. Supplementary Figure 4b shows the histogram of correlation coefficients. Evidently, majority of the images are poorly correlated, indicating that the modes extend over different links in the network.

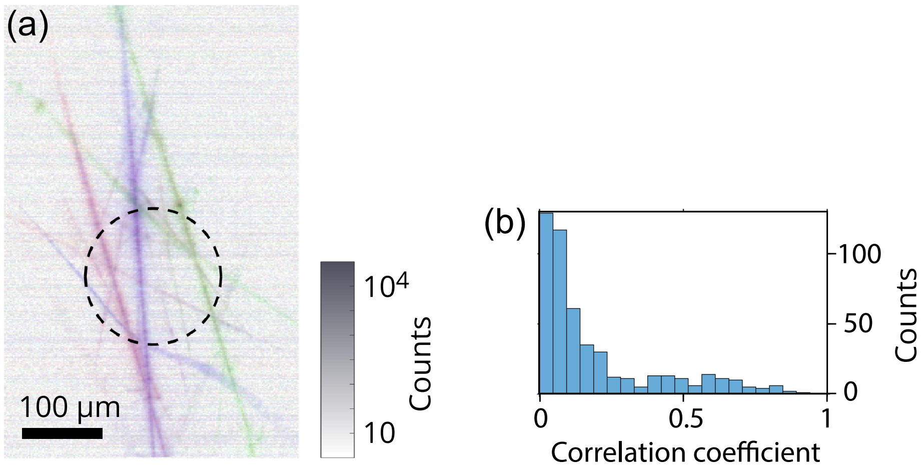

Supplementary Figure 4. **Comparison of different lasing modes in the nanophotonic network**. (a) Composite image formed by overlaying the hyperspectral images in Figure 2a-c of the main manuscript. (b) Correlation between hyperspectral images of 32 different lasing modes.

Supplementary Note 7: Gain length calculation

The gain length of Rh6G (L_g) is estimated from its density in polymer (1% by weight) and its stimulated emission cross-section ($\sigma = 3 \times 10^{-20}$ m^2). The density of the polymer is ~ 1 g cm^{-3} and of Rh6G is 479 g mol^{-1}, so the density of Rh6G in polymer (Π) is:

$$\begin{aligned} \Pi &= 0.01 \times \frac{1 \text{ g cm}^{-3}}{479 \text{ g mol}^{-1}} \times (6 \times 10^{23} \text{ molecules mol}^{-1}) \\ &= 1.25 \times 10^{25} \text{ molecules m}^{-3} \end{aligned} \tag{15}$$

Gain length is thus:

$$\begin{aligned} L_g &= (\Pi \cdot \sigma)^{-1} \\ &= \frac{1}{1.25 \times 10^{25} \text{ m}^{-3} \times 2 \times 10^{-20} \text{ m}^2} \\ &= 4 \text{ μm} \end{aligned} \tag{16}$$

However, this estimate is a best case scenario as it neglects losses and assumes perfect confinement of the mode inside the fibre. By taking into account the overlap of the fundamental mode with the fibre, which varies from 0.4 – 0.8 for fibre diameters between 200–500 nm, the gain length is estimated to be 5–10 μm.

Supplementary Note 8: Sensitivity to changes in refractive index

The calculations of wavelength shift due to refractive index perturbation were performed on a Buffon's graph (Supplementary Figure 5a) of similar dimensions to the networks studied experimentally. A global change in the refractive index across all the links shifts all the lasing modes by the same amount. The shift is proportional to the change in refractive index as shown in Supplementary Figure 5b; the sensitivity is 570 nm per refractive index unit (RIU). Taking into account the linewidth of the lasing modes and the resolution of the spectrometer (0.05 nm), gives a figure of merit (FOM) of 1×10^4 and refractive index detection limit of 1×10^{-4}.

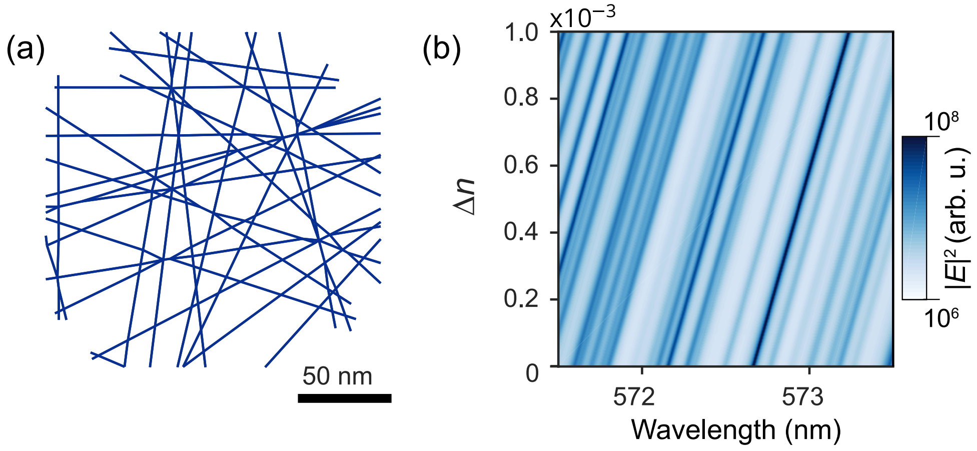

Supplementary Figure 5. **Wavelength shift due to global change in refractive index**. (a) The Buffon's graph used for calculations of wavelength shift due to refractive index perturbation. This graph is quite similar to the networks studied experimentally in terms of its size and topology. It is 200 μm in diameter, and has 157 nodes, 350 links, average degree of 4.5, average link length of 15 μm and a total link length of 4.834 mm. (b) Wavelength shift versus relative index shift globally across the network.
