# Supplementary material for: A nanophotonic laser on a graph
Source: Nat Commun. 2019 Jan 15;10:226. doi: 10.1038/s41467-018-08132-7 (PMC6333791; doi:10.1038/s41467-018-08132-7)
Supplement: Supplementary file 3 — Laser Reporting Summary [file 41467_2018_8132_MOESM3_ESM.pdf]

nature research

Corresponding author(s): Dr Riccardo Sapienza

Lasing Reporting Summary

Nature Research wishes to improve the reproducibility of the work that we publish. This form is intended for publication with all accepted papers reporting claims of lasing and provides structure for consistency and transparency in reporting. Some list items might not apply to an individual manuscript, but all fields must be completed for clarity.

For further information on Nature Research policies, including our data availability policy, see Authors & Referees.

Experimental design

Please check: are the following details reported in the manuscript?

1. Threshold

Plots of device output power versus pump power over a wide range of values indicating a clear threshold

☒ Yes ☐ No — Shown in Fig 1f of the main manuscript

2. Linewidth narrowing

Plots of spectral power density for the emission at pump powers below, around, and above the lasing threshold, indicating a clear linewidth narrowing at threshold

☒ Yes ☐ No — Shown in Fig 1e and Fig 2d of the main manuscript

Resolution of the spectrometer used to make spectral measurements

☒ Yes ☐ No — See Methods.

3. Coherent emission

Measurements of the coherence and/or polarization of the emission

☐ Yes ☒ No — Coherence and polarization were not measured. Polarization could have been measured if our setup/sample was configured/designed so that light emitted end on from the network fibers could be analyzed.

4. Beam spatial profile

Image and/or measurement of the spatial shape and profile of the emission, showing a well-defined beam above threshold

☐ Yes ☒ No — Far-field top-view image shown in Fig 1b. A well-defined beam cannot be seen as the image is of light scattering out of plane from the network. A well-defined beam could be observed if the light emitted out from the fibers could be imaged end on.

5. Operating conditions

Description of the laser and pumping conditions
Continuous-wave, pulsed, temperature of operation

☒ Yes ☐ No — See Methods.

Threshold values provided as density values (e.g. W cm^{-2} or J cm^{-2}) taking into account the area of the device

☒ Yes ☐ No — See Results Paragraph 3.

6. Alternative explanations

Reasoning as to why alternative explanations have been ruled out as responsible for the emission characteristics
e.g. amplified spontaneous, directional scattering; modification of fluorescence spectrum by the cavity

☒ Yes ☐ No — Yes they have been ruled out.

7. Theoretical analysis

Theoretical analysis that ensures that the experimental values measured are realistic and reasonable
e.g. laser threshold, linewidth, cavity gain-loss, efficiency

☒ Yes ☐ No — Yes, we estimated the threshold by modeling. The estimated values were consistent with our experimental values.

8. Statistics

Number of devices fabricated and tested

☒ Yes ☐ No — More than 16 different networks were tested.

Statistical analysis of the device performance and lifetime (time to failure)

☒ Yes ☐ No — Stability of lasing discussed in Supplementary Note 5.
